# Impact of expiratory muscle strength training on swallowing function in Parkinson’s disease: insights from high-resolution videomanometry

**DOI:** 10.1016/j.clinsp.2026.101167

**Published:** 2026-09-26

**Authors:** Fernanda Cristina de Oliveira Rocha, Charles Henrique Dias Marques, Eponina Maria Oliveira Lemme, Luiz João Abrahão‐Júnior

**Affiliations:** a Medical College of Universidade Federal do Rio de Janeiro, Rio de Janeiro, RJ, Brazil; b Digestive Motility Laboratory, Gastroenterology Division of Hospital Universitário Clementino Fraga Filho, Universidade Federal do Rio de Janeiro, Rio de Janeiro, RJ, Brazil

**Keywords:** Manometry, Parkinson disease, Deglutition disorders, Breathing exercises, Rehabilitation

## Abstract

**Introduction:**

Expiratory Muscle Strength Training (EMST) is used for the rehabilitation of oropharyngeal dysphagia in patients with Parkinson's Disease (PD).

**Materials & methods:**

Twenty individuals (16-men) with PD underwent High-Resolution Videomanometry (HRVM) before, during, and after training.

**Results:**

Participants’ mean age was 68.2 ± 8.5 years, with PD staging corresponding to Hoehn and Yahr stage 2 (1.0–3.6). Maximum Expiratory Pressure (MEP) was 79.2 ± 24 cmH_2_O. After training, there was an increase in laryngeal elevation and total displacement (10 mL nectar; *r* = 0.46, 95% CI [0.8, 8.8], p = 0.044 and *r* = 0.53, 95% CI [1.5, 5.9], p = 0.018, respectively). In addition, Intrabolus Pressure (IBP) increased during uncontrolled swallowing (nectar; *r* = 0.56, 95% CI [4.9, 18.6], p = 0.017). Moderate direct and inverse correlations were observed. A direct correlation was found between the Upper Esophageal Sphincter (UES) opening size and age (10 mL nectar; p = 0.037), and between laryngeal anteriorization and stage (10 mL nectar; p = 0.012). An inverse correlation was observed between UES opening size and MEP (10 mL pudding; p = 0.020). A direct correlation was observed between UES relaxation time and stage (10 mL nectar; p = 0.051), and between IBP during uncontrolled swallowing and stage (p = 0.008). The nadir of UES relaxation during uncontrolled swallowing showed a direct correlation with MEP (p = 0.031) and an inverse correlation with stage (p = 0.005).

**Conclusion:**

EMST influences laryngeal displacement. Disease stage, age, and MEP are relevant parameters that should be analyzed during PD rehabilitation. HRVM is a comprehensive tool for evaluating the impacts of EMST both during execution and on swallowing function in patients with PD.

## Highlights

• High-Resolution Videomanometry (HRVM) provides a complete analysis of deglutition.
• Expiratory Muscle Strength Training (EMST) impacts the entire aerodigestive tract.
• Acute changes in laryngeal movement were found after EMST in Parkinson’s disease.
• Disease stage, age and maximum expiratory pressure are relevant for therapy.
• The findings of this study require controlled and longitudinal confirmation.

## Introduction

Expiratory Muscle Strengthening Training (EMST) has presented a new perspective for the rehabilitation of Oropharyngeal Dysphagia (OPD) since its proposal in 2002.^1^ Initially, it aimed to strengthen the muscles involved in respiratory function, and subsequent vocal benefits were also observed due to increased subglottic air pressure. Over the years, its effects on swallowing have gained prominence through observations of the impact of exercise on laryngeal function and the improvement of voluntary coughing in cases of OPD, especially in Parkinson's Disease (PD).^2^ Literature proposes its application in different causes of OPD; however, the population with the greatest rehabilitation success and the most extensively studied is individuals with PD.

OPD in PD comprises a set of alterations, including the following: reduced tongue strength and mobility, reduced laryngeal displacement, reduced reflex cough associated with silent aspiration, reduced pharyngeal constriction, and dysfunction of the Upper Esophageal Sphincter (UES).^3^, ^4^, ^5^, ^6^, ^7^, ^8^ Such alterations can cause oral and/or pharyngeal residue and laryngeal penetration and/or aspiration of the bolus during oral ingestion, which, in the long term, result in clinical impacts such as reduced oral intake, malnutrition, dehydration, bronchoaspiration pneumonia, recurrent hospitalizations, and death.^4^^,^^9^ Because PD is a neurodegenerative disease that affects the indirect descending pyramidal pathway, the onset and progression of OPD are gradual. Therefore, OPD is generally expected to be more severe in advanced stages, since in these cases, the impacts of dysphagia are more aggressive, and the risks associated with oral nutrition are more evident.

However, nowadays, the differential in OPD rehabilitation for this population is precisely professional awareness that dysphagia is already present in the early stages of the disease, even in the presence of low self-perception of signs and symptoms.^10^ PD is the second most common neurodegenerative disease worldwide, most frequently affecting males aged ≥ 65-years.^11^ Furthermore, in these cases, OPD is the main cause of hospitalization and death in more advanced stages of the disease.^12^ Motor rehabilitation in Parkinson's generally proposes that individuals remain in movement from the moment of diagnosis through targeted physical exercise to maintain strength and movement quality. In the same context, speech therapy clinics propose personalized exercises for OPD rehabilitation in the early stages of the disease.

Therefore, it is highly relevant to conduct studies on the rehabilitation of OPD in this population, especially regarding the application of devices that have already proven effective, such as EMST. However, gaps exist in the description of training effects, especially in relation to changes in the larynx, pharynx, and esophagus during active expiration and swallowing immediately after training. A complete description of this process through imaging examinations aims to broaden clinicians’ understanding of indications and possible contraindications for rehabilitation training. High-Resolution Videomanometry (HRVM) is an instrumental method of swallowing assessment that combines Videofluoroscopic Swallowing Study (VFSS) and High-Resolution Manometry (HRM), which, when performed simultaneously, allows qualitative and quantitative analysis of swallowing by measuring dynamic events in association with swallowing pressures. Therefore, this study aimed to describe the immediate effect of EMST on the swallowing of individuals with PD and to describe the impacts observed during expiratory tasks through HRVM.

## Materials & methods

### Inclusion and exclusion criteria

This cross-sectional study was conducted at the Digestive Motility Laboratory of the Gastroenterology Service of the Clementino Fraga Filho University Hospital (HUCFF) of the Federal University of Rio de Janeiro (UFRJ) between December 2018 and December 2022. This study was conducted in accordance with the STROBE Statement. Individuals diagnosed with PD undergoing speech-language therapy participated in the study. The total sample consisted of 20 individuals recruited from the Orofacial Motricity Outpatient Clinic of the Speech-Language Pathology Service of HUCFF of UFRJ and through the Extension Project “Parkinson's Speech-Language Pathology UFRJ.” Participants with potential eligibility for the study were invited as they arrived at the outpatient clinic for care. The study was approved by the Research Ethics Committee of HUCFF of UFRJ (CAAE n° 92868918.4.0000.5257), and all volunteers signed a written informed consent form. The inclusion criteria were: Age ≥ 40-years, both genders; a diagnosis of PD with disease staging according to the Hoehn & Yahr Scale; and ability to understand the study objectives and tasks requested by the researcher. Autoperception of dysphagia symptoms was not considered a criterion for inclusion because it was not relevant to the main objective of the study. The exclusion criteria were: a previous diagnosis of cognitive and/or linguistic deficits; a current diagnosis of severe lung disease; a prior medical restriction on performing lung function tests; a diagnosis of obesity or malnutrition; a diagnosis of or ongoing treatment for head and neck cancer; a history of other neurological diseases that may affect swallowing; pregnancy; and use of medications that interfere with swallowing. Participants were asked to interrupt the prior therapy and medication for PD a week before the exam.

#### Variables studied

Participants underwent an assessment of Maximum Expiratory Pressure (MEP) using an analog manometer (scale 0 to +250 cmH_2_O). For the assessment, each participant remained seated in a chair at a 90° angle at the hip joint. The participant was then instructed to inhale deeply through the mouth, reaching total lung capacity. Upon reaching this capacity, the participant was instructed to manually signal to the researcher and then, through a simple verbal command, was asked to perform a maximal expiratory effort. At this point, the researcher positioned the device, closed the device orifice, and measured the MEP.^13^ This test was repeated three times, and the average MEP value in cmH_2_O was obtained. Next, HRVM was performed before and after expiratory training to verify the immediate impact of this exercise on swallowing.

HRVM consisted of VFSS and HRM performed simultaneously. Initially, the individual was positioned in a special chair for a radiological swallowing examination, as described by Costa (2009).^14^ The videomanometric examination began after topical nasal anesthesia (xylocaine gel; Lidogel®Neoquímica) and the introduction of the HRM catheter (SierraScientific, Los Angeles, USA). Manometric catheter consists of 32 solid-state pressure sensors spaced 1 cm apart and positioned from the soft palate to the stomach. The positioning of the sensors was monitored using a computer with ManoView Z® Analysis software (Medtronic, v2.0.1). After positioning, the probe was fixed to prevent exteriorization. All images were synchronized, recorded at 29.97 frames per second, and then digitized using a computer.

VFSS was captured using a Philips C-arm, with an image intensifier positioned to obtain the following field-of-view limits in the left lateral/profile view: the lips as the anterior limit, the cervical spine as the posterior limit, the hard palate and upper nasal cavity as the upper limit, and the bifurcation of the airway and esophagus at the level of the 7^th^ cervical vertebra as the lower limit, allowing visualization of the UES (Fig. 1). Swallowing was assessed using liquid contrast (barium sulfate solution), according to service's routine protocol, with the following consistencies: original (100% concentration, similar to nectar), honey, and pudding. The consistency was modified by adding the food thickener Resource Thicken Up Clear® (Nestlé Brazil) to the contrast. The contrast in the original consistency (nectar) was offered in a volume of 10 mL with an uncontrolled swallow. For the adapted consistencies, honey and pudding, a fixed volume of 10 mL was maintained. A 250-mL graduated beaker and a 10 mL spoon were used as utensils. The evaluation comprised four different aliquots: 10 mL of nectar consistency administered by spoon, uncontrolled swallowing from a graduated beaker, and 10 mL each of honey and pudding consistency administered by spoon. Each aliquot was offered once during the pre- and post-training evaluations, totaling eight aliquots per participant. The following parameters were analyzed using VFSS: application of the Penetration-Aspiration Scale (PAS);^15^ measurement of hyolaryngeal complex displacement during swallowing in millimeters (mm), including anteriorization, elevation, and total hyoid bone displacement; and measurement of UES opening size during swallowing in millimeters (mm). The software VirtualDub® (developed by Avery Lee, version 1.10.4.0) was used for frame-by-frame analysis and frame selection, and ImageJ® (developed at the National Institutes of Health and Laboratory for Optical and Computation Instrumentation at University of Wisconsin, version 1.53e) was used to extract the videofluoroscopic parameters. All analysis were done by the authors (F.C.O.R., C.H.D.M. and L.J.A.) through a random distribution of exams. All analysts were blinded to the participants’ clinical information’s and evaluation moment (before or after intervention). Fig. 1 illustrates laryngeal displacement and UES opening size during swallowing using VFSS.

**Fig. 1:**
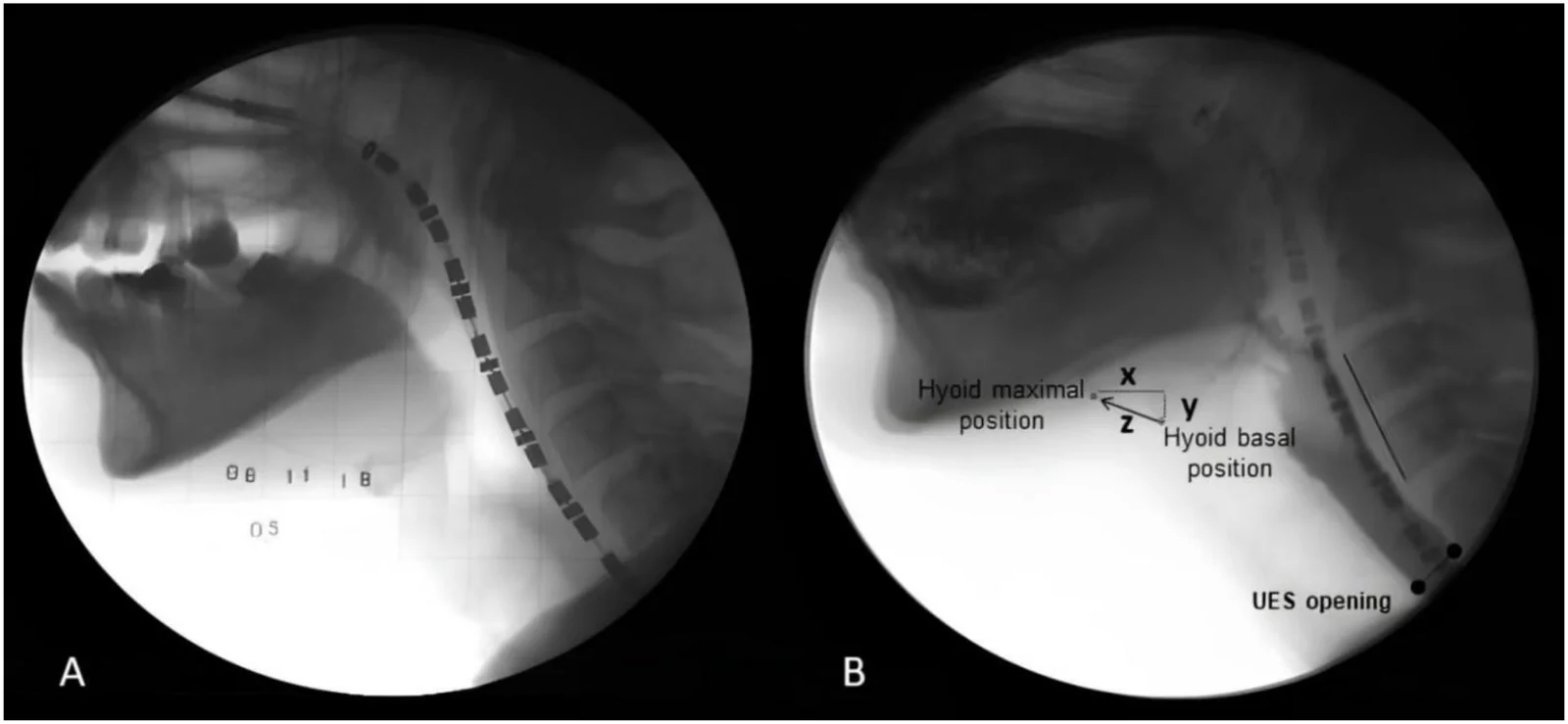
Visual composition of the parameters studied in VFSS. (A) Calibration grid (2 × 2 cm); (B) Recording of the uncontrolled aliquot swallowing; x, anteriorization; y, elevation; z, total displacement; UES, Upper Esophageal Sphincter.

The following parameters^16^ (Fig. 2) were analyzed using HRM with ManoView Z® software: UES basal size (mm); UES basal pressure (mmHg); pharyngeal contraction peak (PCP, mmHg); intrabolus pressure (IBP, mmHg); Nadir, defined as the minimum pressure at the beginning of UES relaxation coinciding with PCP (mmHg), referred to as nadir at PCP (Nadir PCP); Nadir, defined as the minimum pressure during UES relaxation (mmHg), referred to as nadir during UES relaxation (Nadir UES); UES relaxation duration (s).

**Fig. 2:**
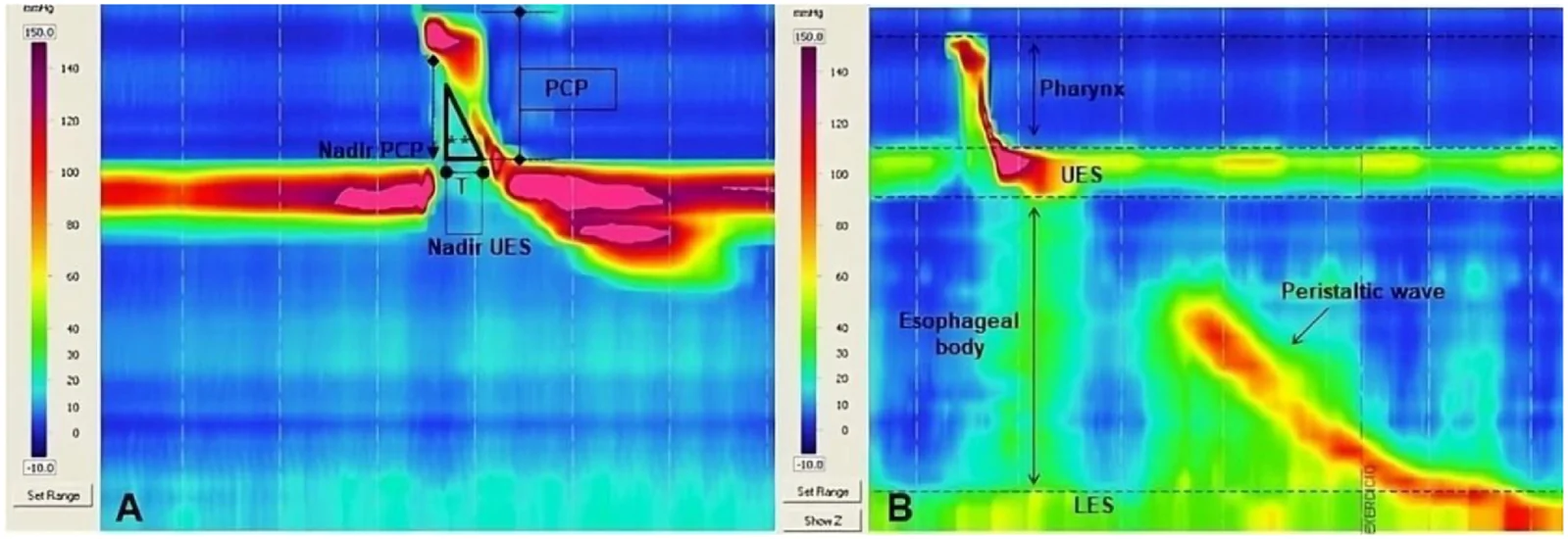
Visual composition of the swallowing pressure parameters studied through HRM. (A) Pharyngeal HRM; (B) Pharyngoesophageal HRM; PCP, Pharyngeal Contraction Peak; Nadir PCP, Nadir at Pharyngeal Contraction Peak; Nadir UES, Nadir during UES relaxation; **, Intrabolus pressure; T, UES relaxation time; LES, Lower Esophageal Sphincter.

After the swallowing assessment, while maintaining the HRM catheter, the participant underwent EMST by performing the expiratory exercise with the EMST 150® device calibrated at 75% of MEP. The participant was instructed to inhale deeply via the oral cavity and then exhale forcefully into the device to displace the internal membrane and open the pressure valve. Five series of five expirations each were applied, with 1-minute intervals between series, totaling 25 expirations, which is the number of repetitions proposed by Sapienza (2012) for a training day.^17^ The videofluoroscopic parameters extracted in this stage during the expiratory task were elevation, anteriorization, and total hyoid displacement. Manometric parameters were maximum pressure in the soft palate, pharynx, and proximal esophagus; UES basal pressure; and UES size.

Fig. 3 illustrates the training visualization through HRVM. During expiration, the anterosuperior displacement of the hyolaryngeal complex occurs in parallel with the velopharyngeal contraction and expansion of the entire pharynx, including the piriform recesses, while the UES remains closed. Simultaneously, a pressure increase was observed, reflecting muscle contraction in the regions of the soft palate, pharynx, UES, and proximal esophagus. The following image shows the regions quantitatively evaluated.

**Fig. 3:**
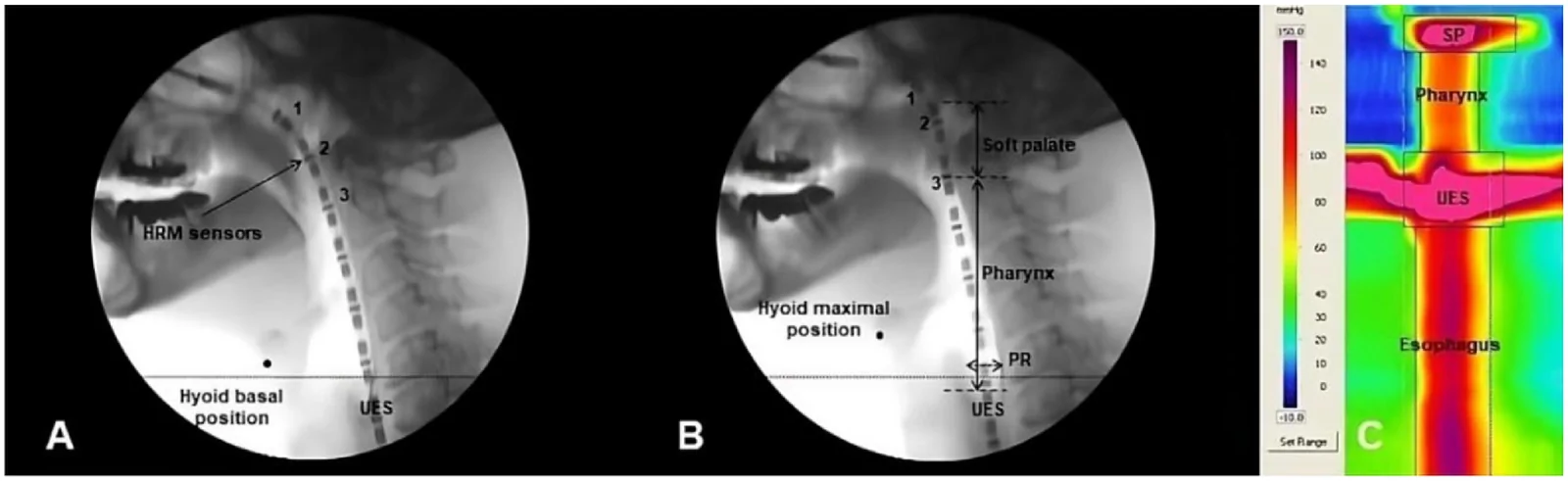
Visual composition of the changes observed during expiration through HRVM. (A) Reveals the basal moment, (B) Reveals the impact during exercise, and (C) Reveals manometric changes in the pharynx and esophagus during exercise. HRM sensors (1, 2, and 3) move upward during soft palate occlusion in image B. HRM, High-Resolution Manometry; UES, Upper Esophageal Sphincter; PR, Piriform Recess; SP, Soft Palate.

Fig. 4 shows the regions of the aerodigestive tract visualized using HRM during expiration. In this image, muscle contraction in each region is identified by a color change, also referred to as heating. Each region (soft palate, pharynx, and esophageal body) transitions from a state of no contraction (rest) to active contraction during expiration. Exceptionally, in the regions of the UES and lower esophageal sphincter (LES), positive basal pressure at rest was maintained and tended to increase during expiration. In this study, LES pressure changes were not quantified because such visualization was not possible in all participants owing to variation in their height and limitations in catheter length.

**Fig. 4:**
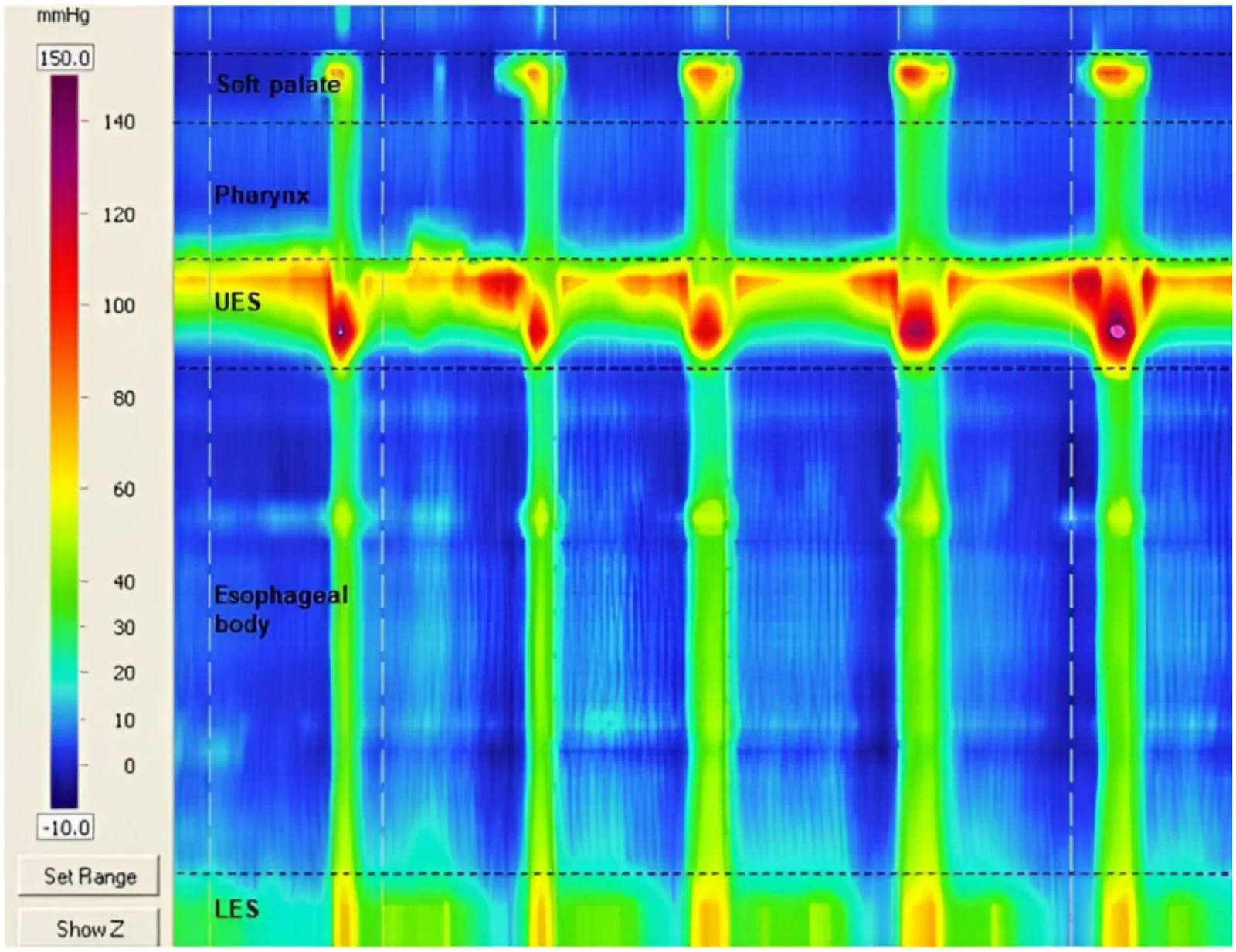
Complete visualization of the aerodigestive tract during exercise using HRM. UES, Upper Esophagel Sphincter; LES, Lower Esophageal Sphincter.

### Statistical analysis

All data were tabulated individually. Descriptive analysis presented the data in tables using appropriate measures of central tendency and dispersion, as well as frequencies and percentages (%) for categorical variables. The inferential analysis consisted of the following methods: changes in HRM and VFSS variables between pre- and post-training were assessed using the Wilcoxon signed-rank test, and associations between numerical clinical variables and the delta values of manometry and VFSS variables were analyzed using Spearman’s correlation coefficient (r_s_). A prior analysis was performed to verify the normality of the variables. For this purpose, the Shapiro-Wilk test was used together with graphical histogram analysis. Because the variables presented a non-Gaussian distribution, nonparametric tests were used. Normally distributed data were expressed as mean and standard deviation, whereas non-normally distributed data were presented as median and interquartile range (Q1–Q3). Categorical data in the sample characterization were expressed as frequencies and percentages. The significance level was set at 5%. In addition to statistical significance, the effect size of the pairwise comparisons performed using the Wilcoxon signed-rank test was estimated by calculating the ratio of the absolute value of the standardized statistic (*Z*) to the square root of the number of valid pairs (*r* = |Z|/√N). The magnitude of the effect was interpreted according to Cohen's criteria, classified as small (*r* ≈ 0.10), moderate (*r* ≈ 0.30), or large (r ≥ 0.50). 95% Confidence Intervals (95% CI) for the difference between time points were estimated using the bootstrap resampling method. Statistical analyses were performed using SPSS (version 26).

## Results

### Primary outcomes

Twenty elderly patients with primary PD participated in this study. The mean age of the group was 68.2 ± 8.5 years, and the majority were male (16 participants). The mean MEP of the group was 79.2 ± 24.0 cmH_2_O. The studied group presented a median stage equivalent to grade 2.0 (1.0–3.6), which is considered an early stage of PD with bilateral manifestation of the disease but without balance deficit. Regarding comorbidities, the following were identified during screening: history of cerebral aneurysm resection (n = 1, 5%), transient ischemic attack (n = 1, 5%), heart failure (n = 5, 25%), use of cardiac pacemaker (n = 2, 10%), controlled systemic arterial hypertension (n = 8, 40%), controlled diabetes mellitus (n = 3, 15%), history of pulmonary tuberculosis (n = 1, 5%), history of botulinum toxin application in the salivary gland for sialorrhea treatment (n = 2, 10%), and history of COVID-19 infection (n = 1, 5%). Previously reported health problems did not interfere with study conduct. Table 1 presents the characteristics of the total sample.

**Table 1:** Characteristics of the total sample.

Variables	Total sample (n = 20)
Age (years)	68.2 ± 8.5
Gender (Male)	16 (80.0%)
Weight (kg)	70.1 ± 11.9
Height (m)	1.68 ± 0.11
MEP (cmH_2_O)	79.2 ± 24.0
Staging (grade)	2.0 (1.0–3.6)

Data are expressed as mean and standard deviation, median (interquartile range: Q1–Q3), and frequency and percentage (%) for categorical data.

Twenty examinations were performed, including VFSS and HRM. The following results represent changes from pre- to post-intervention. During sample analysis, two HRM examinations were lost due to equipment failure; therefore, HRM evaluation was performed based on 18 examinations. All VFSS examinations (20) were performed and analyzed. Regarding the aliquot size of each uncontrolled swallow, 11 measurements were recovered at both pre- and post-intervention times, and nine were lost due to equipment failure during data recording. The mean value representing the group with recovered samples was obtained. There was variability in the volume of this aliquot, ranging from 19.54 ± 6.50 mL pre-intervention to 22.72 ± 6.84 mL post-intervention. Through VFSS, all participants scored grade 1 according to the PAS, indicating absence of laryngeal penetration and/or aspiration in all aliquots at pre- and post-intervention. Table 2 compares laryngeal displacement measurements during swallowing across different aliquots at pre- and post-intervention using VFSS. In the 10 mL nectar-consistency aliquot there was a significant increase in laryngeal elevation (*r* = 0.46, 95% CI [0.8, 8.8], p = 0.044), with a moderate effect size and the difference’s estimate of approximately 5.4 in the confidence interval, when comparing pre-intervention (median = 10.8 mm) with post-intervention (median = 16.5 mm). In addition, in this aliquot also were found a significant increase in total laryngeal displacement (*r* = 0.53, 95% CI [1.5, 5.9], p = 0.018) with a large effect size and the difference’s estimate of approximately 3.9 in the confidence interval, when comparing pre-intervention (median = 18.9 mm) with post-intervention (median = 23.4 mm). There was no statistically significant variation in the other videofluoroscopic variables. Fig. 5, Fig. 6 illustrate increases in laryngeal elevation and total laryngeal displacement with the 10-mL nectar-consistency aliquot at pre- and post-intervention. During the expiratory exercise with resistance, laryngeal displacement was observed through VFSS, with laryngeal anteriorization of 3.24 mm (-0.32–4.96), elevation of 7.13 mm (2.89–9.35), and total displacement of 8.52 mm (5.25–10.03). Fig. 7 compares laryngeal displacements during swallowing of the 10 mL nectar-consistency aliquot at pre-intervention with those observed during expiratory exercise. It was observed that laryngeal displacement occurred in both conditions; however, during exercise, total displacement (8.52 mm, 5.25–10.03) occurred to a lesser extent compared with swallowing (18.9 mm, 16.3–23.8). During swallowing, greater laryngeal anteriorization was observed (13.6 mm, 8.1–16.8) compared with elevation (10.8 mm, 7.3–19.4), whereas during exercise, this mechanism was reversed: laryngeal elevation (7.13 mm, 2.89–9.35) exceeded anteriorization (3.24 mm, -0.32–4.96).

**Table 2:** Laryngeal displacement through VFSS pre- and post-intervention.

Videofluoroscopy Swallow Study												
Tests	Anteriorization (mm)			Elevation (mm)			Total displacement (mm)			UES opening size (mm)		
	Pre	Post	p-value	Pre	Post	p-value	Pre	Post	p-value	Pre	Post	p-value
Nectar (10 mL)	13.6 (8.1–16.8)	15.4 (10.4–18.3)	0.08	10.8 (7.3–19.4)	16.5 (12.6–20.8)	0.044^a^	18.9 (16.3–23.8)	23.4 (20.8–25.3)	0.018^a^	8.7 (7.0–10.4)	9.4 (7.9–10.2)	0.15
Nectar (uncontrolled swallow)	12.3 (10.6–17.9)	13.4 (9.5–17.2)	0.52	12.5 (7.0–19.8)	13.5 (9.5–16.6)	0.97	18.9 (15.0–23.5)	19.9 (13.7–26.8)	0.65	9.7 (8.8–12.5)	9.6 (8.4–11.8)	0.66
Honey (10 mL)	14.6 (10.8–15.8)	13.4 (8.9–16.9)	0.85	12.3 (9.1–18.8)	14.8 (10.6–20.6)	0.16	18.5 (16.1–22.8)	21.2 (15.2–24.9)	0.18	9.9 (8.0–11.1)	9.5 (9.0–11.7)	0.33
Pudding (10 mL)	14.2 (9.1–18.5)	15.2 (10.6–7.6)	0.70	15.8 (9.5–20.6)	14.9 (11.1–19.5)	0.88	20.6 (17.1–26.5)	23.1 (16.9–25.6)	0.75	10.8 (9.0–12.6)	10.1 (8.7–11.4)	0.62

Data are expressed as median (interquartile range: Q1–Q3) and were compared using the Wilcoxon signed-rank test.

a p < 0.05 was considered statistically significant.

**Fig. 5:**
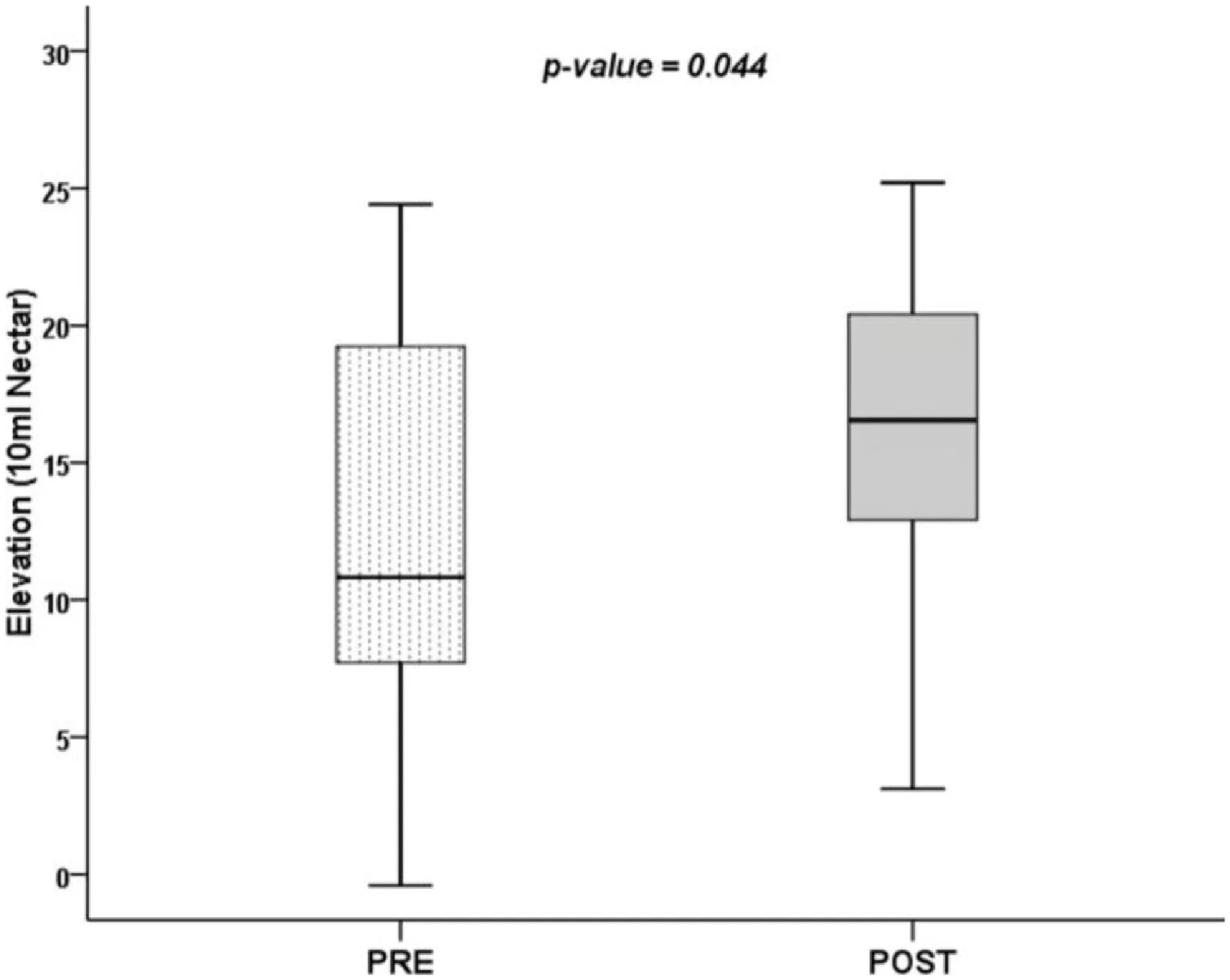
Laryngeal elevation (mm) in the 10 mL nectar-consistency aliquot at pre- and post-intervention.

**Fig. 6:**
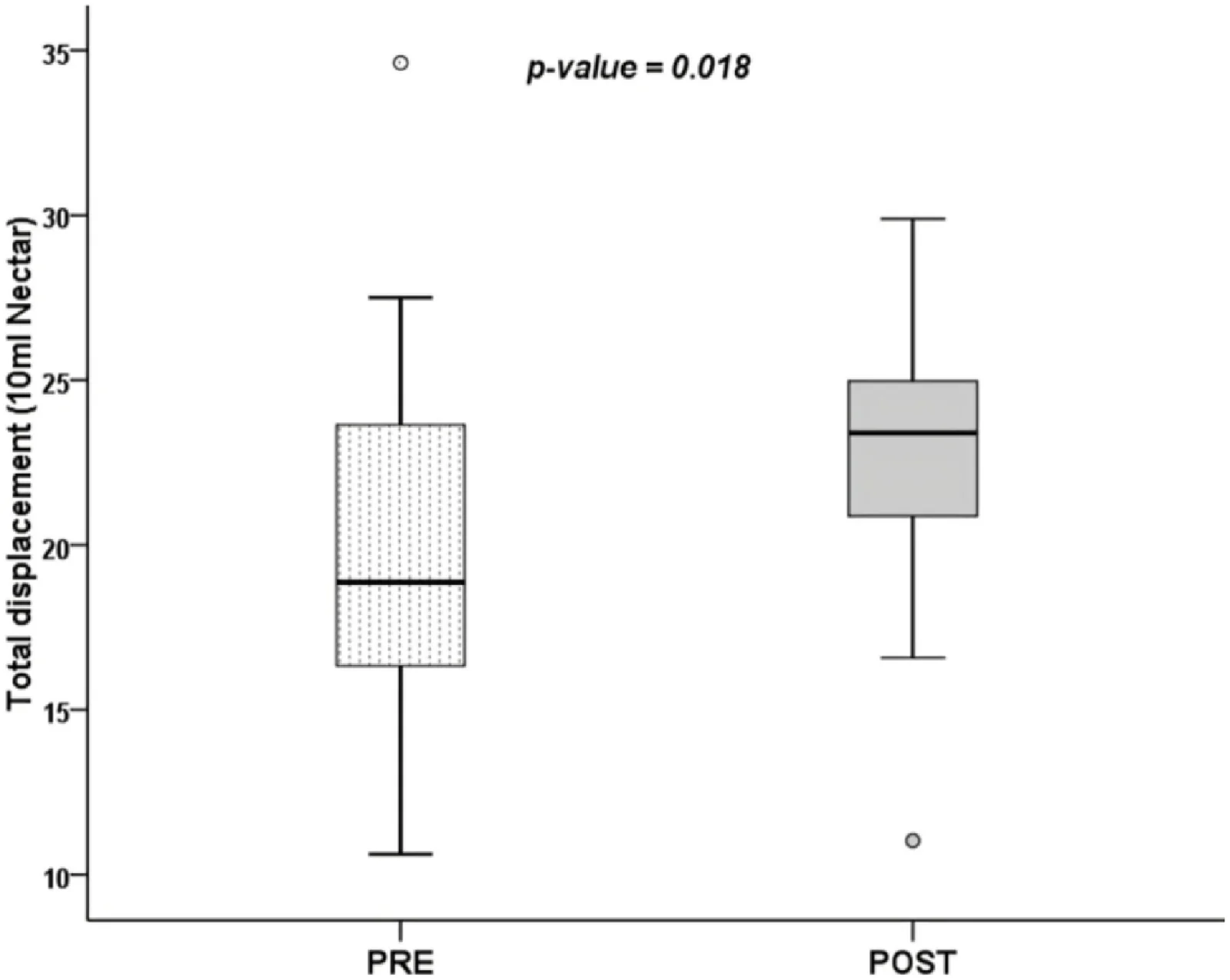
Total laryngeal displacement (mm) in the 10 mL nectar-consistency aliquot at pre- and post-intervention.

**Fig. 7:**
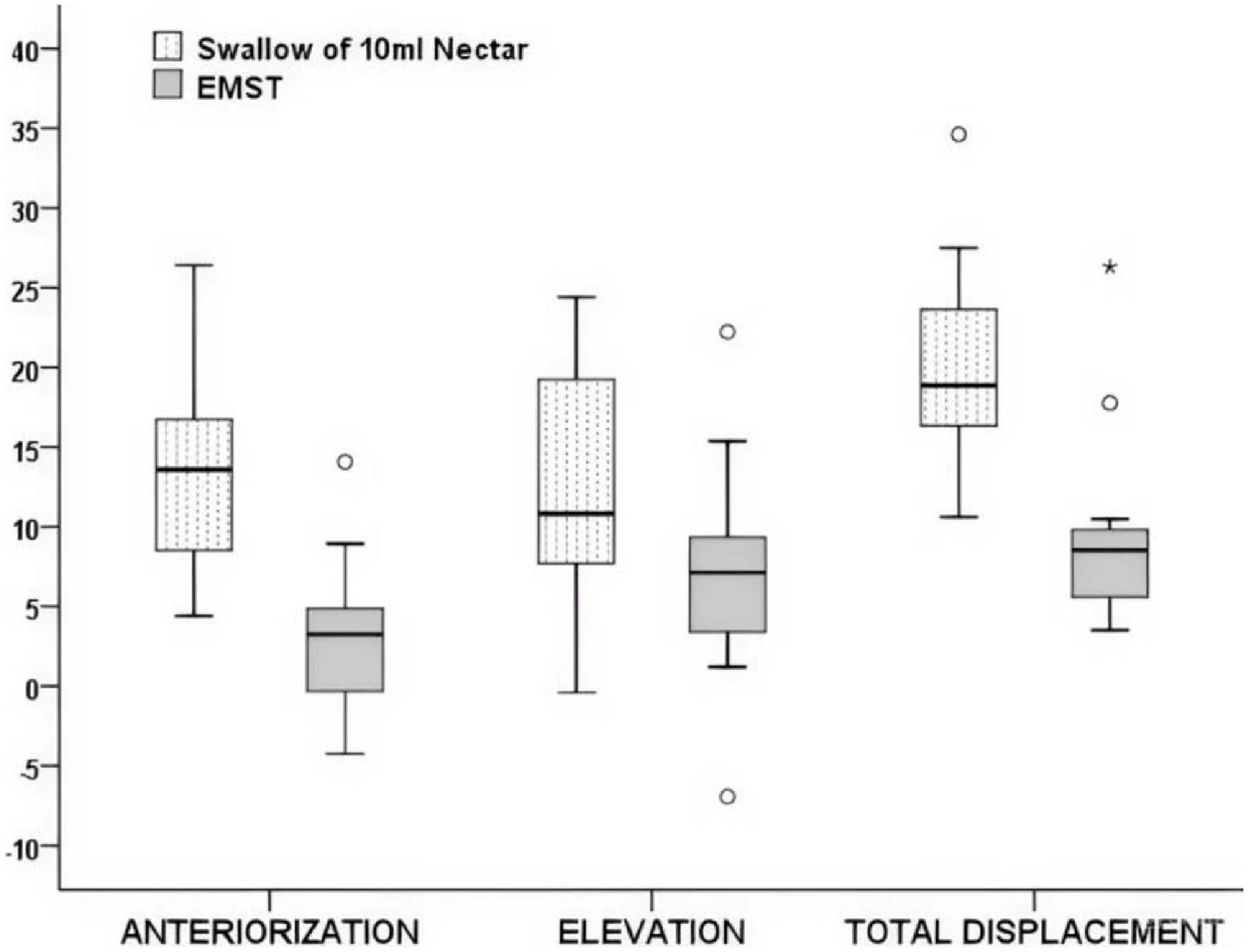
Laryngeal displacement (mm) during swallowing and exercise.

Table 3 compares HRM variables at pre- and post-intervention. PCP remained high at both time points and in all consistencies. After training, a tendency toward an increase in this variable was observed in the 10 mL honey-consistency aliquot (p = 0.08). IBP remained high at pre- and post-intervention. In addition, a significant increase in IBP was observed in the uncontrolled nectar-consistency swallow (*r* = 0.56, 95% CI [4.9, 18.6], p = 0.017) from pre-intervention (median = 17.3 mmHg) to post-intervention (median = 27.3 mmHg). In relation to p value, were found a large effect size and the difference’s estimate showed a value of approximately 10.8 in the confidence interval.

**Table 3:** HRM variables at pre- and post-intervention.

High Resolution Manometry															
Tests	PCP (mmHg)			IBP (mmHg)			Nadir PCP (mmHg)			Nadir UES (mmHg)			UES relaxation time (s)		
	Pre	Post	p-value	Pre	Post	p-value	Pre	Post	p-value	Pre	Post	p-value	Pre	Post	p-value
Nectar (10 mL)	301 (238– 334)	262 (210 –341)	0.35	21.7 (16.0 – 40.4)	18.8 (12.1 – 41.2)	0.35	11.4 (7.6–16.2)	11.4 (7.2– 15.7)	0.92	1.60 (0–4.98)	1.50 (0–7.96)	0.25	0.50 (0.39–0.55)	0.50 (0.45–0.60)	0.06
Nectar (uncontrolled swallow)	235 (208 – 369)	245 (183 – 335)	0.74	17.3 (14.2 – 26.2)	27.3 (14.1 – 39.6)	0.017^a^	9.5 (7.5–12.0)	10.4 (9.0– 15.3)	0.26	1.28 (0–7.25)	1.30 (0–5.60)	0.97	0.43 (0.30–0.51)	0.50 (0.30–0.60)	0.60
Honey (10 mL)	235 (199 – 400)	234 (179 – 372)	0.53	27.4 (17.2 – 35.2)	20.3 (13.1 – 31.7)	0.23	9.3 (6.9–13.3)	12.8 (6.2– 15.7)	0.08	0.38 (0– 6.25)	0 (0–4.94)	0.86	0.40 (0.38–0.50)	0.43 (0.30–0.50)	0.90
Pudding (10 mL)	237 (203 – 347)	248 (193 – 358)	0.91	20.4 (13.8 – 32.4)	18.9 (15.6 – 27.7)	0.45	10.0 (7.6– 13.6)	10.4 (8.2– 15.0)	0.28	0.60 (0– 5.30)	0.10 (0– 3.35)	0.48	0.42 (0.30– 0.50)	0.45 (0.38– 0.50)	0.40

Data are expressed as median (interquartile range: Q1–Q3) and compared using the Wilcoxon signed-rank test.

PCP, Pharyngeal Contraction Peak; IBP, Intrabolus Pressure; UES, Upper Esophageal Sphincter; Nadir PCP, Nadir at the PCP; Nadir UES, Nadir during UES opening.

a Statistical significance, p < 0.05.

The nadir corresponding to the PCP remained high, and the nadir corresponding to UES opening reached a lower value (median = 0) only in the 10 mL honey-consistency aliquot post-intervention. The highest value observed for the UES relaxation time was 0.5 seconds (median) at pre- and post-intervention. Regarding this variable, no change was observed in the UES relaxation time post-intervention, with a tendency for equal pre- and post-intervention values in the 10 mL nectar-consistency aliquot (p = 0.06). There were no statistically significant variations in the other manometric variables.

### Exploratory outcomes

In the manometric evaluation of the pharynx at rest, no change in UES basal pressure was observed from pre-intervention (42.2 mmHg, 22.9–61.3) to post-intervention (35.0 mmHg, 22.0–58.3) (p = 0.12). Similarly, no change in UES basal size was observed from pre-intervention (2.8 cm, 2.38–3.65) to post-intervention (2.65 cm, 2.38–3.55) (p = 0.92). However, an increase in pharyngeal and esophageal pressure was observed during expiratory exercise. During exercise, UES basal pressure reached 71.5 mmHg (47–101) and basal size reached 4.2 cm (4.0–5.1). Furthermore, there was an increase in pressurization in the soft palate (143 mmHg, 88–216), pharynx (78.1 mmHg, 61–91), and proximal esophagus (96.6 mmHg, 66–106).

We found correlations between variables such as staging, age, and MEP and the deltas of different swallowing parameters. There was a moderate, significant direct correlation between the degree of staging and the delta of laryngeal anteriorization for the 10 mL nectar-consistency aliquot (*r*_s_ = 0.55; p = 0.012), as well as between staging and the delta of UES relaxation time for the same aliquot (*r*_s_ = 0.47; p = 0.051). In addition, a moderate, significant direct correlation was observed between the degree of staging and the delta of IBP for the uncontrolled swallow in nectar consistency (*r*_s_ = 0.60; p = 0.008). However, despite elevated IBP with increasing stage, a significant, moderate inverse correlation was observed between staging and the delta of nadir during UES relaxation for the uncontrolled swallow in nectar consistency (*r*_s_ = -0.62; p = 0.005). Age also influenced UES opening size. There was a significant, moderate direct correlation between age and the delta of UES opening size for the 10 mL nectar-consistency aliquot (*r*_s_ = 0.47; p = 0.037).

In addition, a significant, moderate direct correlation was observed between MEP and the delta of the nadir during UES relaxation in the uncontrolled swallow in the nectar consistency (*r*_s_ = 0.51; p = 0.031). Finally, a significant, moderate inverse correlation was observed between MEP and the delta of UES opening size for the 10 mL pudding-consistency aliquot (*r*_s_ = -0.51; p = 0.020). The raw survey data is consolidated in Supplementary Material.

## Discussion

PD is a neurodegenerative disease prevalent worldwide. Population growth, aging, and increased disease duration are factors that contribute to its rising prevalence. Regarding incidence, studies have indicated a higher prevalence in males, in whom disease progression is more aggressive compared with females.^18^ In this study, the majority of the sample consisted of elderly males, consistent with the literature. In addition, the mean age of the sample was over 65 years, as reported in previous studies.^19^ To perform EMST, it was necessary to measure the participants' MEP. According to Costa (2010), there is a significant difference between MEP values according to gender, and the authors proposed normal values for the Brazilian population according to age group.^20^ In this study of elderly participants with PD, the mean MEP value for both genders was 79.2 ± 24.0 cmH_2_O, which is below the values reported in healthy individuals in the Brazilian population in this age group (60–69 years).

Regarding swallowing, the severity of OPD in PD is associated with worsening motor symptoms, especially in terms of increased rigidity and tremor.^21^ Although the diagnosis of OPD is broad and does not depend solely on changes in the pharyngeal phase of swallowing, its severity is directly associated with the presence of laryngeal aspiration. In this context, the literature indicates that changes in the pharyngeal phase of swallowing can be observed even in the early stages of the disease.^22^ Unlike previous reports, participants in this study, whose disease manifestations were in the early stages, did not present laryngeal penetration and/or aspiration during HRVM at pre- and post-intervention. However, this finding is insufficient to exclude the presence of OPD. One possible explanation is that participants had already undergone speech therapy before the study, with interventions generally aimed at maximizing airway protection mechanisms.

VFSS demonstrated that EMST immediately increases laryngeal displacement during swallowing, particularly vertical movement. A study by Troche et al. (2010) showed that EMST applied to individuals with PD is an important tool for OPD rehabilitation, leading to improved PAS scores by increasing hyolaryngeal complex displacement during swallowing.^23^ However, the present study shows a specific impact on vertical laryngeal displacement during post-training swallowing. Since laryngeal displacement was also measured during training, anterosuperior displacement was characterized mainly by elevation, exceeding anteriorization. After training, the same laryngeal pattern was immediately observed during swallowing.

Therefore, EMST generates an acute increase in total laryngeal displacement during swallowing; however, its main impact is on vertical movement. During pre-training swallowing, anteriorization exceeded elevation; during training, this mechanism was reversed. Laryngeal elevation is closely related to occlusion of the laryngeal vestibule, whereas laryngeal anteriorization has a greater influence on UES opening during swallowing.^24^^,^^25^ Therefore, individuals with PD in the early stage may exhibit compensatory mechanisms that favor UES opening, including greater laryngeal anteriorization compared with elevation, and EMST appears to compensate this mechanism.

For years, manometric studies have described disturbances in swallowing in individuals with PD. Ali et al. (1996) reported different swallowing alterations related to UES behavior in patients with PD. Among these are incomplete UES relaxation in terms of pressure, reduced UES opening diameter, and elevated IBP.^26^ IBP increases as UES compliance to food bolus passage decreases. Thus, under normal conditions of UES relaxation and sufficient reduction in its internal pressure, IBP is expected to be low. In this study, elevated IBP was observed at pre- and post-intervention, with a further increase observed post-intervention, particularly in the uncontrolled swallow. Nollet et al. recently reported that an increase in food bolus volume is directly associated with increased IBP during swallowing in young, healthy individuals, independent of viscosity.^27^ Therefore, although the present study evaluated the immediate effects on swallowing, the increase in IBP observed only in the uncontrolled swallow may be related to the increase in aliquot volume during post-training evaluation. In the post-intervention period, participants tended to fill the oral cavity with a larger volume of liquid contrast, possibly reflecting increased confidence in swallowing after performing the exercise.

At the same time, high PCP values were maintained at pre- and post-intervention. The literature reveals that with advancing age, normal individuals may exhibit reduced UES compliance to food bolus passage due to neuronal modification of and decreased distensibility of the cricopharyngeal muscle. In response, compensatory pharyngeal mechanisms may occur to facilitate the passage of the food bolus through the UES, including increased pharyngeal effort, similar to the effortful swallowing maneuver.^28^ Thus, in PD, elevated PCP in the early stage is interpreted as a compensatory mechanism that facilitates food bolus conduction through the pharynx and promotes UES opening. Therefore, during rehabilitation, it is important to include targeted interventions for this segment in the early stages of PD.

The nadir related to PCP and UES relaxation remained elevated at pre- and post-intervention. The elevation of these parameters was considered to be associated with the increased IBP and PCP values observed during the examination. Regarding UES size and basal pressure, the literature reports values of approximately 47.30 ± 15.58 mmHg for basal pressure and 2–4 cm for the basal size of the UES.^29^ These findings are consistent with those of this study. At pre-exercise, UES basal pressure was 42.2 mmHg (22.9–61.3) and basal size was 2.8 cm (2.38–3.65). At post-exercise, basal pressure was 35.0 mmHg (22.0–58.3) and basal size was 2.65 cm (2.38–3.55). However, during exercise, UES basal pressure was 71.5 mmHg (47–101) and UES basal size was 4.2 cm (4.0–5.1). This finding demonstrates stretching and toning of the UES during expiratory training, similar to the impact generated by vocal maneuvers, such as hyperacute sound and the prolonged b technique, which have been previously described in the literature.^7^^,^^30^

Hutcheson et. al. (2017) reported changes in the aerodigestive tract during expiratory exercise. In that study, two healthy individuals underwent simultaneous electromyography and HRM. An increase in velopharyngeal sealing and electromyographic activity of the soft palate and pharynx was observed with increasing device load. In contrast, thyroarytenoid muscle activity decreased during exercise, likely due to glottic opening during expiration.^31^ Thus, the present study brings advances in research regarding the evaluation of expiratory training by demonstrating the impacts on the UES and esophagus. It was observed that contraction occurred not only in the soft palate (143 mmHg, 88–216) and pharynx (78.1 mmHg, 61–91) occurs, but also in the UES and proximal esophagus (96.6 mmHg, 66–106). The literature reveals that contraction of the proximal esophagus during expiratory maneuvers occurs through activation of the esophageal branch of the recurrent nerve, preventing air entry into the esophagus.^32^ In this study, we hypothesize that pressurized expiration induces a similar esophageal contraction mechanism. However, additional electromyographic or neurophysiologic evidence is necessary to confirm this hypothesis.

Regarding the variables studied, significant correlations were observed between disease stage, age, and MEP and the deltas of different swallowing parameters, consistent with HRVM results. Although all participants were in the early stages of PD, variation in staging indicated that, as stage increased, there was a greater tendency toward laryngeal anteriorization and prolonged UES opening time. At the same time, advancing stage was also associated with increased IBP. This finding indicates that disease progression may increase resistance to food bolus passage through the UES, leading to the emergence of compensatory pharyngeal mechanisms to overcome this resistance, as previously explained.^28^ Regarding the nadir of UES opening, advancing PD stage was associated with lower nadir values, reaffirming the efficiency of the compensatory mechanisms in the studied group. Notably, a previous study that used HRM to assess the UES in PD reported changes in UES contraction and opening, especially in more advanced stages.^33^ Therefore, even in the early stages of PD, the worsening of OPD and UES dysfunction is influenced by disease stage, and this aspect needs to be carefully considered during swallowing rehabilitation in this population.

Age also influenced UES behavior during swallowing. Notably, older age was associated with greater UES opening, which reinforces the existence of compensatory mechanisms. Therefore, in PD, UES dysfunction may be influenced not only by disease stage but also by aging. According to a study by Ding et al.^34^ advanced age and disease progression are the main risk factors for OPD development in patients with PD, and each of these parameters independently influences OPD. In contrast, MEP, which reflects the intensity of active expiration in this study, was also found to influence UES behavior. It was observed that the stronger the active expiration of the individual with PD, that is, the higher the MEP, the greater the nadir of UES relaxation. In other words, expiratory exercise performed with a greater intensity was associated with a reduced UES admittance. Additionally, increased MEP was associated with a reduced UES opening, particularly with pudding consistency. Under normal conditions, greater UES opening is expected with increasing food bolus viscosity.^27^ However, participants with greater MEP, who are generally healthier and with less motor impairment, presented smaller UES opening with more viscous boluses. Although the small sample size limits the interpretation and generalizability of these findings, the observed correlations highlight the relationship between UES function, disease stage, age, and MEP in PD.

The present study provides a detailed and novel evaluation of the acute impacts of expiratory training on swallowing in individuals with PD. An in-depth investigation of this therapeutic technique may enable more precise clinical indication based on a clearer understanding of its effects on the aerodigestive tract and UES function, thereby helping to prevent its indiscriminate use. It’s important to mention that the training was applied in patients in the early stages of PD and these findings can’t be extrapolated to patients in advanced stages (grade 4 and 5 of Hoehn and Yahr Scale), where dysphagia generally is more severe, and the response to EMST may differ.

The assessment of OPD has evolved over the years. Through HRVM, it is possible to specifically identify which aspect of swallowing is dysfunctional. Consequently, overly broad therapies can prove time-consuming and even ineffective. Guided by the principle of efficacy, an exercise must achieve its main purpose. So, what matters is not how many muscles the exercise engages, but whether it specifically addresses the patient's needs. EMST has shown promise as a specific training method for restoring laryngeal displacement in the early stages of PD. However, it should be noted that during the training occurs an intermittent pressure increasing in the UES. As the disease progresses, complications related to UES function tend to arise. Therefore, in such cases, it is advisable to prioritize exercises proven to facilitate UES relaxation, either alongside or instead of EMST.

This study has some limitations, including the small sample size and the absence of a control group. However, relevant studies have been conducted using similar designs and sample sizes.^35^^,^^36^ In addition, completing training was not performed prospectively, as this was a cross-sectional study. However, this design provides an opportunity to clarify some possible acute effects of exercise. Recent evidence suggests that EMST leads to greater changes in maximum hyoid displacement after 4-weeks of training.^37^ This study demonstrated that similar changes may occur acutely after exercise.

We assume that medication for PD and its variation status, as well as HRM catheter placement are relevant topics in deglutition evaluation; however, further research is necessary to clarify their influence on function. Although the images processing were done by a specialist’s team, the absence of a reliability testing during exams analysis could be a study limitation and future studies including this measurement are encouraged.

Despite these limitations, the results provide important elucidation regarding the mechanisms involved in the execution of the exercise, how EMST alters physiology during use and its immediate therapeutic effect. Longitudinal studies with larger sample sizes and control groups are required to assess the retention of benefits and to corroborate these findings.

## Conclusion

This study demonstrates that HRVM is an important tool for assessing swallowing and therapeutic techniques in individuals with PD. The effects of EMST are variable and depend on the individual's baseline status. EMST is associated with an acute increase in laryngeal displacement and IBP during swallowing, as well as increased UES residual pressure and basal size during the expiratory task. Disease stage, age, and MEP are clinical characteristics that are closely associated with UES function in patients with PD. Although EMST has important clinical applicability, it should not be indicated indiscriminately in the rehabilitation of OPD in PD. However, we found that EMST can be used as an acute facilitation maneuver before meals mainly for patients with laryngeal elevation deficits. Therefore, further studies are warranted.

## Declaration of generative AI

It was not used generative AI and AI-assisted technologies in the writing process in the manuscript.

## Authors’ contributions

Fernanda Cristina de Oliveira Rocha designed the study together with Charles Henrique Dias Marques, Eponina Maria Oliveira Lemme, and Luiz João Abrahão‐Júnior; Fernanda Cristina de Oliveira Rocha, Charles Henrique Dias Marques, and Luiz João Abrahão‐Júnior conducted the research and analyzed the data; Fernanda Cristina de Oliveira Rocha drafted the manuscript; all authors carefully revised the manuscript.

## Declaration of competing interest

The authors declare no conflicts of interest.

## Data Availability

We declare that all relevant data are available within the manuscript. The raw data from this study have been made available in the manuscript and in the Supplementary Material.
