# Supplementary material for: Impact of expiratory muscle strength training on swallowing function in Parkinson’s disease: insights from high-resolution videomanometry
Source: Clinics (Sao Paulo). 2026 Sep 26;81:101167. doi: 10.1016/j.clinsp.2026.101167 (PMC13636329; doi:10.1016/j.clinsp.2026.101167)
Supplement: Supplementary file 1 [file mmc1.docx]

**CLINICS-D-26-01240_Supplementary Material**

| **Characterization of the total sample** | | | | | | | **HRM during exercise** | | | | | **VFSS during exercise** | | |
| --- | --- | --- | --- | --- | --- | --- | --- | --- | --- | --- | --- | --- | --- | --- |
| **N** | **Gender** | **Age (years)** | **Weight (kg)** | **Height (m)** | **MEP (cmH_2_O)** | **Staging (grade)** | **UES Baseline pressure (mmHg)** | **UES Baseline Size (cm)** | **Soft palate pressure (mmHg)** | **Pharingeal pressure (mmHg)** | **Proximal esophagus pressure (mmHg)** | **Anteriorization (mm)** | **Elevation (mm)** | **Total Displacement (mm)** |
| 1 | Male | 59 | 90,0 | 1,81 | 60,0 | 1 | 154,020 | 4,020 | 100,280 | 75,840 | 96,700 | -0,708 | 10,464 | 10,488 |
| 2 | Male | 69 | 68,0 | 1,69 | 97,0 | 2 | 39,280 | 5,740 | 161,040 | 86,160 | 110,420 | -3,917 | 4,917 | 6,286 |
| 3 | Male | 63 | 75,0 | 1,82 | 114,0 | 1 | 152,300 | 4,960 | 215,160 | 94,900 | 119,220 | -4,255 | 2,128 | 4,758 |
| 4 | Male | 66 | 67,0 | 1,66 | 120,0 | 2 | 83,440 | 4,240 | 227,500 | 91,560 | 101,020 | 4,651 | 9,302 | 10,400 |
| 5 | Male | 70 | 73,0 | 1,65 | 103,3 | 1 | 63,840 | 4,140 | 124,920 | 50,960 | 45,000 | 6,400 | 1,200 | 6,512 |
| 6 | Male | 67 | 60,0 | 1,73 | 73,0 | 1 | 42,680 | 5,520 | 147,280 | 70,940 | 74,100 | 14,074 | 22,222 | 26,304 |
| 7 | Male | 63 | 62,0 | 1,72 | 83,0 | 2 | 48,900 | 4,140 | 138,880 | 82,480 | 105,100 | 8,929 | 15,357 | 17,764 |
| 8 | Male | 65 | 82,0 | 1,75 | 70,0 | 1 | 107,980 | 5,480 | 78,000 | 68,840 | 76,060 | 0,816 | 10,204 | 10,237 |
| 9 | Male | 77 | 65,8 | 1,68 | 80,0 | 2 | 84,020 | 4,680 | 163,180 | 85,840 | 75,380 | 3,043 | 1,739 | 3,505 |
| 10 | Male | 83 | 70,0 | 1,62 | 70,0 | 4 | 67,840 | 4,360 | 77,920 | 63,720 | 96,400 | 0,851 | 8,936 | 8,977 |
| 11 | Female | 63 | 53,0 | 1,60 | 50,0 | 2,5 | 40,040 | 3,300 | 92,740 | 80,340 | 67,380 | 5,926 | 5,926 | 8,381 |
| 12 | Male | 55 | 82,0 | 1,78 | 110,0 | 1 | 95,980 | 4,600 | 273,480 | 110,360 | 107,100 | -0,320 | 9,360 | 9,365 |
| 13 | Female | 64 | 80,0 | 1,58 | 65,0 | 2 | 48,800 | 4,200 | 91,500 | 46,740 | 58,560 | -1,167 | 8,583 | 8,662 |
| 14 | Male | 64 | 52,0 | 1,75 | 50,0 | 4 | 50,780 | 4,000 | 79,480 | 39,880 | 35,980 | 4,306 | 2,361 | 4,910 |
| 15 | Male | 61 | 78,0 | 1,70 | 80,0 | 2 | 201,360 | 4,000 | 219,720 | 91,020 | 102,080 | 4,170 | 8,426 | 9,401 |
| 16 | Male | 81 | 65,0 | 1,60 | 58,3 | 4 | 75,140 | 3,020 | 155,140 | 75,640 | 98,280 | 3,444 | 8,333 | 9,017 |
| 17 | Male | 72 | 85,8 | 1,79 | 113,3 | 1,5 | 98,300 | 5,620 | 240,300 | 136,580 | 147,940 | -0,327 | 4,571 | 4,583 |
| 18 | Female | 89 | 47,0 | 1,39 | 33,3 | 5 | 13,020 | 3,680 | 63,320 | 46,440 | 61,240 | 3,822 | -6,933 | 7,917 |
| 19 | Male | 63 | 64,0 | 1,74 | 80,0 | 1,5 | * | * | * | * | * | 5,069 | 4,653 | 6,881 |
| 20 | Female | 70 | 83,0 | 1,53 | 74,0 | 4 | * | * | * | * | * | 1,118 | 4,471 | 4,608 |

| **HRM before intervention** | | | | | | | | | | | | | | | | | | | | | |
| --- | --- | --- | --- | --- | --- | --- | --- | --- | --- | --- | --- | --- | --- | --- | --- | --- | --- | --- | --- | --- | --- |
| **UES Baseline pressure (mmHg)** | **UES Baseline Size (cm)** | **Nectar (10ml)** | | | | | **Nectar (uncontrolled swallow)** | | | | | **Honey (10ml)** | | | | | **Pudding (10ml)** | | | | |
|  |  | **PCP (mmHg)** | **IBP (mmHg)** | **NADIR PCP (mmHg)** | **NADIR UES (mmHg)** | **UES relaxation time (s)** | **PCP (mmHg)** | **IBP (mmHg)** | **NADIR PCP (mmHg)** | **NADIR UES (mmHg)** | **UES relaxation time (s)** | **PCP (mmHg)** | **IBP (mmHg)** | **NADIR PCP (mmHg)** | **NADIR UES (mmHg)** | **UES relaxation time (s)** | **PCP (mmHg)** | **IBP (mmHg)** | **NADIR PCP (mmHg)** | **NADIR UES (mmHg)** | **UES relaxation time (s)** |
| 74,700 | 2,800 | 341,100 | 32,350 | 16,100 | 12,350 | 0,550 | 226,400 | 18,500 | 10,800 | 15,900 | 0,400 | 232,600 | 50,900 | 12,700 | 16,400 | 0,600 | 178,400 | 66,700 | 14,700 | 25,400 | 0,500 |
| 17,500 | 2,100 | 249,600 | 17,600 | 16,400 | 0,000 | 0,500 | 426,050 | 22,300 | 22,800 | 11,000 | 0,270 | 517,600 | 25,800 | 14,900 | 12,500 | 0,500 | 412,350 | 21,550 | 24,400 | 9,950 | 0,350 |
| 93,500 | 4,000 | 245,000 | 44,100 | 11,600 | 5,500 | 0,500 | 227,300 | 30,800 | 4,700 | 0,000 | 0,400 | 253,600 | 35,400 | 6,700 | 0,000 | 0,500 | 213,100 | 23,700 | 4,500 | 0,900 | 0,400 |
| 43,400 | 2,600 | 331,700 | 17,900 | 12,800 | 4,800 | 0,400 | 558,700 | 15,400 | 9,500 | 2,700 | 0,300 | 387,000 | 12,100 | 8,400 | 0,000 | 0,400 | 471,900 | 12,200 | 8,900 | 5,100 | 0,150 |
| 23,200 | 3,600 | 268,700 | 12,900 | 0,000 | 0,000 | 0,500 | 232,400 | 10,400 | 2,300 | 0,000 | 0,500 | 249,800 | 11,300 | 0,000 | 0,000 | 0,500 | 213,100 | 18,700 | 0,000 | 0,000 | 0,500 |
| 36,600 | 2,800 | 169,830 | 35,360 | 11,160 | 4,430 | 0,360 | 164,200 | 37,800 | 11,400 | 1,500 | 0,450 | 228,400 | 28,100 | 9,800 | 6,100 | 0,500 | 172,300 | 33,750 | 9,200 | 0,000 | 0,430 |
| 52,100 | 2,500 | 216,000 | 39,100 | 17,000 | 1,900 | 0,200 | 222,300 | 20,200 | 13,200 | 0,000 | 0,300 | 218,200 | 28,800 | 6,900 | 0,000 | 0,200 | 206,700 | 30,700 | 8,600 | 0,000 | 0,300 |
| 53,400 | 2,800 | 307,950 | 15,050 | 6,850 | 2,700 | 1,100 | 221,350 | 14,250 | 9,400 | 1,750 | 0,450 | 217,550 | 11,100 | 15,450 | 2,850 | 0,300 | 395,750 | 11,150 | 10,200 | 6,000 | 0,500 |
| 61,100 | 3,500 | 328,350 | 47,900 | 7,850 | 1,300 | 0,350 | 350,550 | 40,250 | 7,950 | 0,500 | 0,350 | 449,450 | 47,100 | 7,450 | 0,350 | 0,400 | 212,400 | 22,700 | 4,100 | 0,000 | 0,220 |
| 30,000 | 2,400 | 421,700 | 82,900 | 4,600 | 0,000 | 0,400 | 164,500 | 44,300 | 6,300 | 0,000 | 0,500 | 190,800 | 26,800 | 1,700 | 0,000 | 0,500 | 250,100 | 33,400 | 3,700 | 0,300 | 0,500 |
| 61,800 | 2,500 | 314,500 | 58,600 | 8,900 | 8,300 | 0,500 | 544,300 | 16,000 | 11,600 | 11,100 | 0,600 | 528,000 | 54,300 | 18,200 | 6,700 | 0,400 | 343,500 | 58,100 | 16,400 | 5,900 | 0,600 |
| 107,000 | 3,900 | 313,800 | 32,000 | 16,800 | 0,000 | 0,700 | 269,000 | 3,300 | 0,000 | 0,000 | 0,800 | 328,400 | 28,000 | 4,350 | 0,000 | 0,400 | 358,300 | 12,460 | 12,700 | 0,000 | 0,460 |
| 19,000 | 2,300 | 247,800 | 16,000 | 15,400 | 9,500 | 0,600 | 238,400 | 22,250 | 12,950 | 6,200 | 0,550 | 237,300 | 18,100 | 10,300 | 11,800 | 0,500 | 231,000 | 13,300 | 14,500 | 4,200 | 0,600 |
| 59,500 | 1,700 | 476,000 | 16,000 | 8,900 | 2,200 | 0,200 | 450,700 | 14,400 | 8,700 | 10,400 | 0,300 | 198,000 | 17,600 | 8,400 | 0,000 | 0,300 | 254,700 | 14,500 | 12,400 | 0,000 | 0,300 |
| 41,000 | 3,900 | 532,550 | 19,700 | 21,650 | 0,000 | 0,550 | 348,600 | 14,100 | 34,600 | 0,000 | 0,600 | 437,300 | 21,000 | 34,800 | 5,400 | 0,300 | 326,000 | 19,200 | 10,300 | 2,900 | 0,400 |
| 21,900 | 2,100 | 207,200 | 16,500 | 12,500 | 0,000 | 0,400 | 169,450 | 24,700 | 11,650 | 1,050 | 0,400 | 195,650 | 35,100 | 8,800 | 0,500 | 0,400 | 192,500 | 32,000 | 9,800 | 0,000 | 0,400 |
| 27,200 | 3,800 | 294,250 | 23,750 | 6,900 | 0,000 | 0,400 | 314,900 | 13,400 | 9,000 | 0,000 | 0,250 | 170,300 | 34,500 | 9,700 | 0,000 | 0,400 | 243,000 | 18,600 | 8,700 | 0,000 | 0,230 |
| 15,600 | 3,400 | 203,200 | 10,800 | 8,700 | 0,500 | 0,500 | 165,300 | 15,700 | 8,950 | 1,850 | 0,450 | 199,900 | 16,050 | 9,850 | 0,400 | 0,500 | 193,600 | 13,900 | 13,300 | 2,100 | 0,500 |

| **HRM after intervention** | | | | | | | | | | | | | | | | | | | | | |
| --- | --- | --- | --- | --- | --- | --- | --- | --- | --- | --- | --- | --- | --- | --- | --- | --- | --- | --- | --- | --- | --- |
| **UES Baseline pressure (mmHg)** | **UES Baseline Size (cm)** | **Nectar (10ml)** | | | | | **Nectar (uncontrolled swallow)** | | | | | **Honey (10ml)** | | | | | **Pudding (10ml)** | | | | |
|  |  | **PCP (mmHg)** | **IBP (mmHg)** | **NADIR PCP (mmHg)** | **NADIR UES (mmHg)** | **UES relaxation time (s)** | **PCP (mmHg)** | **IBP (mmHg)** | **NADIR PCP (mmHg)** | **NADIR UES (mmHg)** | **UES relaxation time (s)** | **PCP (mmHg)** | **IBP (mmHg)** | **NADIR PCP (mmHg)** | **NADIR UES (mmHg)** | **UES relaxation time (s)** | **PCP (mmHg)** | **IBP (mmHg)** | **NADIR PCP (mmHg)** | **NADIR UES (mmHg)** | **UES relaxation time (s)** |
| 69,800 | 3,500 | 223,300 | 38,100 | 29,100 | 17,600 | 0,600 | 331,800 | 22,100 | 15,900 | 16,800 | 0,500 | 179,600 | 10,900 | 35,900 | 25,400 | 0,500 | 158,900 | 20,300 | 17,800 | 21,100 | 0,500 |
| 18,200 | 2,200 | 441,500 | 40,900 | 12,600 | 13,500 | 0,500 | 180,900 | 29,000 | 10,400 | 0,900 | 0,200 | 368,100 | 29,100 | 16,100 | 0,000 | 0,300 | 254,600 | 17,800 | 14,300 | 2,400 | 0,600 |
| 79,900 | 2,300 | 274,700 | 7,800 | 8,200 | 6,200 | 0,600 | 255,000 | 10,100 | 6,800 | 0,300 | 0,500 | 241,500 | 12,900 | 14,700 | 0,700 | 0,400 | 197,000 | 10,500 | 10,900 | 0,200 | 0,300 |
| 29,000 | 3,800 | 263,300 | 15,800 | 9,600 | 6,600 | 0,500 | 396,000 | 38,000 | 9,700 | 5,500 | 0,500 | 230,200 | 18,800 | 22,500 | 7,700 | 0,300 | 365,900 | 17,900 | 7,300 | 3,100 | 0,400 |
| 11,800 | 2,900 | 342,100 | 8,500 | 0,000 | 0,000 | 0,500 | 267,300 | 5,900 | 0,000 | 0,000 | 0,300 | 203,200 | 9,700 | 5,700 | 0,000 | 0,600 | 202,600 | 17,900 | 3,250 | 0,000 | 0,550 |
| 35,500 | 3,700 | 202,100 | 25,300 | 18,100 | 1,800 | 0,450 | 147,100 | 29,400 | 26,800 | 1,800 | 0,450 | 145,300 | 27,000 | 13,400 | 0,950 | 0,500 | 182,700 | 14,300 | 12,400 | 1,000 | 0,500 |
| 54,500 | 2,700 | 231,100 | 67,000 | 11,800 | 1,200 | 0,400 | 291,500 | 24,200 | 10,000 | 0,000 | 0,300 | 306,600 | 39,500 | 10,300 | 0,000 | 0,210 | 304,300 | 31,200 | 12,900 | 0,000 | 0,270 |
| 0,000 | 2,600 | 260,950 | 10,400 | 10,650 | 5,150 | 0,450 | 190,700 | 18,100 | 13,500 | 11,600 | 0,500 | 224,000 | 9,900 | 13,000 | 10,300 | 0,500 | 208,700 | 11,100 | 18,300 | 11,800 | 0,500 |
| 34,400 | 2,800 | 319,500 | 45,700 | 9,000 | 0,000 | 0,500 | 468,850 | 60,050 | 6,900 | 2,100 | 0,280 | 451,600 | 44,000 | 5,000 | 0,000 | 0,400 | 466,800 | 29,300 | 9,100 | 0,000 | 0,220 |
| 44,800 | 2,400 | 210,600 | 51,500 | 0,000 | 0,000 | 0,500 | 185,350 | 75,900 | 5,850 | 0,000 | 0,450 | 178,350 | 67,650 | 4,950 | 0,000 | 0,450 | 163,300 | 30,500 | 8,400 | 0,000 | 0,400 |
| 37,000 | 2,100 | 276,000 | 41,900 | 10,900 | 0,800 | 0,500 | 345,000 | 46,900 | 10,300 | 8,200 | 0,600 | 542,100 | 48,300 | 11,300 | 0,000 | 0,500 | 517,200 | 47,000 | 9,800 | 0,000 | 0,500 |
| 110,300 | 2,400 | 411,800 | 11,600 | 3,000 | 0,000 | 0,600 | 359,650 | 13,300 | 24,000 | 0,200 | 0,600 | 383,100 | 13,200 | 6,200 | 0,000 | 0,400 | 355,900 | 12,800 | 9,700 | 0,000 | 0,300 |
| 25,900 | 2,100 | 209,600 | 17,300 | 15,900 | 11,700 | 0,600 | 231,500 | 39,100 | 15,100 | 3,800 | 0,600 | 218,400 | 18,000 | 15,600 | 9,500 | 0,600 | 234,700 | 19,400 | 12,500 | 10,000 | 0,600 |
| 36,000 | 2,400 | 160,900 | 12,200 | 4,200 | 0,000 | 0,400 | 178,900 | 12,900 | 12,600 | 0,000 | 0,300 | 139,400 | 22,200 | 11,600 | 0,000 | 0,300 | 278,000 | 19,500 | 7,400 | 0,000 | 0,400 |
| 27,100 | 4,600 | 447,800 | 15,700 | 15,600 | 0,000 | 0,700 | 330,700 | 14,400 | 10,400 | 0,000 | 0,600 | 488,250 | 19,550 | 12,500 | 4,020 | 0,450 | 400,950 | 16,000 | 17,100 | 2,250 | 0,450 |
| 23,300 | 3,900 | 341,200 | 17,800 | 14,600 | 0,000 | 0,700 | 178,550 | 41,250 | 14,650 | 0,000 | 0,350 | 296,900 | 21,000 | 15,600 | 0,000 | 0,400 | 241,000 | 18,350 | 9,500 | 0,000 | 0,450 |
| 1,000 | 3,500 | 259,800 | 28,700 | 15,800 | 7,400 | 0,400 | 234,800 | 25,600 | 10,300 | 5,900 | 0,500 | 167,400 | 21,800 | 6,100 | 1,900 | 0,300 | 285,800 | 27,100 | 6,700 | 4,100 | 0,400 |
| 72,700 | 2,500 | 158,460 | 19,830 | 14,660 | 9,630 | 0,530 | 184,000 | 35,100 | 22,800 | 1,700 | 0,600 | 238,500 | 14,200 | 18,750 | 0,000 | 0,500 | 148,800 | 20,000 | 17,400 | 0,000 | 0,500 |

| **VFSS before intervention** | | | | | | | | | | | | | | | |
| --- | --- | --- | --- | --- | --- | --- | --- | --- | --- | --- | --- | --- | --- | --- | --- |
| **Nectar (10ml)** | | | | **Nectar (uncontrolled swallow)** | | | | **Honey (10ml)** | | | | **Pudding (10ml)** | | | |
| **Anteriorization (mm)** | **Elevation (mm)** | **Total displacement (mm)** | **UES opening size (mm)** | **Anteriorization (mm)** | **Elevation (mm)** | **Total displacement (mm)** | **UES opening size (mm)** | **Anteriorization (mm)** | **Elevation (mm)** | **Total displacement (mm)** | **UES opening size (mm)** | **Anteriorization (mm)** | **Elevation (mm)** | **Total displacement (mm)** | **UES opening size (mm)** |
| 20,062 | 11,014 | 22,887 | 11,408 | 18,095 | 21,636 | 28,205 | 11,801 | 15,342 | 18,882 | 24,329 | 10,228 | 16,128 | 21,636 | 26,986 | 11,014 |
| 15,417 | 17,500 | 23,322 | 10,000 | 12,083 | 20,417 | 23,724 | 13,750 | 10,000 | 12,500 | 16,008 | 12,083 | 13,750 | 15,000 | 20,349 | 12,500 |
| 7,660 | 10,638 | 13,109 | 9,362 | 11,489 | 14,043 | 18,144 | 9,362 | 11,064 | 14,894 | 18,553 | 8,936 | 8,936 | 17,872 | 19,982 | 9,362 |
| 10,698 | 6,977 | 12,772 | 6,977 | 10,698 | 9,767 | 14,486 | 9,767 | 10,698 | 11,163 | 15,461 | 7,442 | 8,837 | 14,419 | 16,911 | 9,767 |
| 26,400 | 22,400 | 34,623 | 3,600 | 18,400 | 6,800 | 19,616 | 7,600 | 15,600 | 6,000 | 16,714 | 6,400 | 14,400 | 8,800 | 16,876 | 10,800 |
| 17,778 | 6,667 | 18,987 | 7,037 | 18,148 | 7,407 | 19,602 | 12,593 | 15,556 | 11,852 | 19,556 | 10,741 | 19,259 | 8,148 | 20,912 | 8,889 |
| 5,714 | 16,786 | 17,732 | 11,071 | 7,143 | 9,643 | 12,000 | 12,143 | 11,071 | 3,929 | 11,748 | 11,071 | 9,286 | 8,929 | 12,882 | 10,714 |
| 9,388 | 22,041 | 23,957 | 7,755 | 10,612 | 14,286 | 17,796 | 8,571 | 6,531 | 19,184 | 20,265 | 8,980 | 10,204 | 11,020 | 15,019 | 9,796 |
| 15,652 | 22,609 | 27,498 | 7,826 | 20,870 | 20,435 | 29,208 | 9,565 | 19,565 | 26,522 | 32,958 | 10,870 | 23,913 | 16,522 | 29,065 | 12,609 |
| 12,766 | 10,213 | 16,348 | 5,957 | 12,340 | 15,319 | 19,671 | 7,660 | 14,468 | 18,723 | 23,662 | 10,638 | 14,043 | 18,723 | 23,404 | 11,915 |
| 13,704 | 8,889 | 16,334 | 4,074 | 12,222 | 18,148 | 21,880 | 9,630 | 12,963 | 12,593 | 18,072 | 8,148 | 12,593 | 13,704 | 18,611 | 7,037 |
| 4,800 | 19,600 | 20,179 | 15,600 | 8,800 | 3,200 | 9,364 | 15,600 | 6,400 | 10,000 | 11,873 | 14,800 | 4,400 | 17,200 | 17,754 | 14,000 |
| 4,583 | 16,250 | 16,884 | 8,333 | 2,083 | 27,917 | 27,994 | 14,167 | 6,667 | 15,000 | 16,415 | 9,583 | 5,417 | 21,250 | 21,929 | 12,917 |
| 9,583 | 4,583 | 10,623 | 7,083 | 12,083 | 7,917 | 14,446 | 7,083 | 16,250 | 8,750 | 18,456 | 8,750 | 16,250 | 6,667 | 17,564 | 7,083 |
| 16,596 | 8,936 | 18,849 | 9,362 | 18,723 | 12,766 | 22,661 | 9,362 | 15,745 | 11,489 | 19,491 | 11,064 | 19,574 | 17,872 | 26,506 | 12,766 |
| 16,889 | 8,444 | 18,882 | 9,333 | 17,333 | 0,444 | 17,339 | 9,333 | 14,667 | 25,333 | 29,273 | 8,000 | 20,000 | 24,000 | 31,241 | 8,889 |
| 13,469 | -0,408 | 13,476 | 8,980 | 13,469 | 12,245 | 18,203 | 12,245 | 11,837 | 0,816 | 11,865 | 11,837 | 8,980 | 3,673 | 9,702 | 11,837 |
| 17,778 | 4,444 | 18,325 | 4,444 | 16,000 | 4,444 | 16,606 | 4,000 | 16,000 | 5,778 | 17,011 | 5,333 | 20,444 | 13,333 | 24,408 | 6,222 |
| 14,722 | 18,889 | 23,949 | 10,556 | 16,111 | 22,500 | 27,673 | 11,389 | 18,056 | 23,889 | 29,945 | 7,500 | 15,833 | 23,056 | 27,969 | 9,444 |
| 4,412 | 24,412 | 24,807 | 15,294 | 6,765 | 5,000 | 8,412 | 15,882 | 15,882 | 12,059 | 19,942 | 14,412 | 15,294 | 21,471 | 26,361 | 15,588 |

| **VFSS after intervention** | | | | | | | | | | | | | | | |
| --- | --- | --- | --- | --- | --- | --- | --- | --- | --- | --- | --- | --- | --- | --- | --- |
| **Nectar (10ml)** | | | | **Nectar (uncontrolled swallow)** | | | | **Honey (10ml)** | | | | **Pudding (10ml)** | | | |
| **Anteriorization (mm)** | **Elevation (mm)** | **Total displacement (mm)** | **UES opening size (mm)** | **Anteriorization (mm)** | **Elevation (mm)** | **Total displacement (mm)** | **UES opening size (mm)** | **Anteriorization (mm)** | **Elevation (mm)** | **Total displacement (mm)** | **UES opening size (mm)** | **Anteriorization (mm)** | **Elevation (mm)** | **Total displacement (mm)** | **UES opening size (mm)** |
| 15,342 | 18,882 | 24,329 | 9,048 | 17,702 | 25,963 | 31,423 | 8,261 | 14,161 | 21,636 | 25,858 | 8,654 | 15,342 | 18,882 | 24,329 | 10,228 |
| 14,167 | 21,250 | 25,539 | 10,000 | 15,833 | 16,667 | 22,989 | 8,333 | 14,583 | 16,250 | 21,834 | 9,167 | 15,000 | 13,750 | 20,349 | 10,000 |
| 11,064 | 12,340 | 16,574 | 10,213 | 8,936 | 10,213 | 13,570 | 9,787 | 8,511 | 17,872 | 19,795 | 11,915 | 8,511 | 14,468 | 16,786 | 8,936 |
| 10,233 | 13,488 | 16,930 | 9,302 | 15,349 | 9,302 | 17,948 | 9,302 | 10,233 | 21,860 | 24,137 | 11,628 | 13,488 | 10,698 | 17,216 | 8,372 |
| 18,400 | 14,000 | 23,121 | 5,600 | 18,400 | 6,000 | 19,354 | 8,400 | 17,200 | 4,800 | 17,857 | 7,600 | 19,600 | 12,000 | 22,982 | 8,400 |
| 15,556 | 15,185 | 21,739 | 10,000 | 13,333 | 25,556 | 28,825 | 11,481 | 13,704 | 26,667 | 29,982 | 9,259 | 15,926 | 18,519 | 24,425 | 9,630 |
| 10,000 | 16,786 | 19,539 | 10,000 | 7,857 | 11,071 | 13,576 | 12,857 | 10,000 | 7,857 | 12,717 | 11,786 | 11,071 | 3,929 | 11,748 | 10,714 |
| 12,245 | 17,143 | 21,067 | 10,204 | 13,469 | 15,510 | 20,542 | 9,388 | 7,347 | 11,837 | 13,931 | 9,796 | 11,020 | 13,469 | 17,403 | 11,020 |
| 17,391 | 19,565 | 26,177 | 12,174 | 18,696 | 16,522 | 24,950 | 11,739 | 18,696 | 11,304 | 21,848 | 9,130 | 20,000 | 19,130 | 27,676 | 9,565 |
| 17,447 | 24,255 | 29,878 | 7,660 | 15,319 | 14,894 | 21,366 | 6,383 | 19,149 | 14,043 | 23,746 | 8,936 | 17,872 | 14,894 | 23,265 | 8,511 |
| 18,148 | 16,296 | 24,391 | 6,667 | 11,852 | 11,111 | 16,246 | 8,889 | 12,593 | 11,111 | 16,794 | 9,259 | 15,556 | 14,815 | 21,481 | 9,630 |
| 9,600 | 25,200 | 26,967 | 10,800 | 8,000 | 10,800 | 13,440 | 14,800 | 6,800 | 10,400 | 12,426 | 14,800 | 6,000 | 10,800 | 12,355 | 15,600 |
| 4,583 | 23,333 | 23,779 | 10,833 | 5,833 | 28,333 | 28,928 | 13,750 | 8,333 | 17,500 | 19,383 | 10,833 | 2,500 | 25,833 | 25,954 | 13,333 |
| 17,500 | 15,833 | 23,600 | 6,250 | 12,917 | 5,417 | 14,006 | 7,500 | 6,250 | 10,417 | 12,148 | 9,167 | 10,417 | 4,583 | 11,380 | 7,500 |
| 21,702 | 9,362 | 23,635 | 9,362 | 23,830 | 14,043 | 27,660 | 10,213 | 17,021 | 19,574 | 25,940 | 11,064 | 15,745 | 19,574 | 25,121 | 12,766 |
| 22,667 | 4,889 | 23,188 | 8,444 | 11,111 | 12,889 | 17,017 | 8,889 | 21,333 | 12,889 | 24,925 | 8,889 | 20,889 | 15,111 | 25,782 | 11,556 |
| 8,571 | 6,939 | 11,028 | 8,980 | 12,245 | -4,490 | 13,042 | 11,837 | 13,061 | 6,531 | 14,603 | 11,837 | 8,980 | 8,571 | 12,414 | 8,571 |
| 20,444 | 3,111 | 20,680 | 7,556 | 22,667 | 15,111 | 27,242 | 7,556 | 13,333 | 15,556 | 20,488 | 7,556 | 20,000 | 31,556 | 37,360 | 11,111 |
| 18,333 | 23,611 | 29,893 | 9,444 | 15,833 | 19,722 | 25,292 | 11,389 | 16,667 | 27,500 | 32,156 | 11,389 | 16,667 | 27,222 | 31,919 | 10,556 |
| 11,471 | 18,529 | 21,793 | 14,412 | 5,000 | 0,882 | 5,077 | 15,294 | 13,529 | 20,882 | 24,882 | 15,294 | 14,412 | 20,588 | 25,131 | 13,824 |
